# The FIFA 11+ injury prevention program for soccer players: a systematic review

**DOI:** 10.1186/s13102-017-0083-z

**Published:** 2017-11-28

**Authors:** David Sadigursky, Juliana Almeida Braid, Diogo Neiva Lemos De Lira, Bruno Almeida Barreto Machado, Rogério Jamil Fernandes Carneiro, Paulo Oliveira Colavolpe

**Affiliations:** 1 Division of knee Surgery, Clínica Ortopédica Traumatológica – COT., Rua Colmar Americano da Costa, 121, Pituba. Apt 1404C, Salvador, Bahia 41830-600 Brazil; 2 Medical School; Department of Orthopedics, Faculdade de Tecnologia e Ciências – FTC, Salvador, Bahia Brazil

**Keywords:** Injury, Prevention, Soccer

## Abstract

**Background:**

Soccer is one of the most widely played sports in the world. However, soccer players have an increased risk of lower limb injury. These injuries may be caused by both modifiable and non-modifiable factors, justifying the adoption of an injury prevention program such as the Fédération Internationale de Football Association (FIFA) 11+. The purpose of this study was to evaluate the efficacy of the FIFA 11+ injury prevention program for soccer players.

**Methodology:**

This meta-analysis was based on the PRISMA 2015 protocol. A search using the keywords “FIFA,” “injury prevention,” and “football” found 183 articles in the PubMed, MEDLINE, LILACS, SciELO, and ScienceDirect databases. Of these, 6 studies were selected, all of which were randomized clinical trials.

**Results:**

The sample consisted of 6,344 players, comprising 3,307 (52%) in the intervention group and 3,037 (48%) in the control group. The FIFA 11+ program reduced injuries in soccer players by 30%, with an estimated relative risk of 0.70 (95% confidence interval, 0.52–0.93, p = 0.01). In the intervention group, 779 (24%) players had injuries, while in the control group, 1,219 (40%) players had injuries. However, this pattern was not homogeneous throughout the studies because of clinical and methodological differences in the samples. This study showed no publication bias.

**Conclusion:**

The FIFA 11+ warm-up program reduced the risk of injury in soccer players by 30%.

## Background

Soccer is the most popular sport worldwide, with approximately 400 million players in 208 countries, generating approximately 1 trillion US dollars per year [1]. The Fédération Internationale de Football Association (FIFA) estimates that 270 million soccer players are registered worldwide [2, 3]. The Brazilian Football Confederation reports 2.1 million federation athletes and 11.2 million amateur athletes in Brazil, without considering those who play soccer recreationally [4]. However, soccer is a contact sport that requires physical aptitude and the ability to play at high levels of intensity [5]. Therefore, soccer carries a significant risk of injuries for both professional and amateur players, as in the case of most other sports, regardless of age [6].

In addition to causing large financial losses for professional soccer leagues, injuries lead to player withdrawals and decreased team performance at the professional and amateur levels [7]. Recent studies indicate that injuries occur mainly during the first and last 15 minutes of the game, which highlights the importance of an appropriate warm-up and the possible effect of fatigue on players [8].

Epidemiological studies categorize injury severity according to a player’s period of inactivity for better understanding and classification as follows: minimal (1–3 days), medium (4–7 days), moderate (8–28 days), or severe (>28 days) [4–9]. Most injuries (60–90%) occur in the lower limbs, especially the ankle, knee (anterior cruciate ligament), and thigh (quadriceps and hamstrings). These are non-contact injuries [5, 10] that occur without impact in players [11], and include sprains, strains, and contusions that mainly affect the thigh and calf muscles and ankle and knee joints [10, 12]. These are mainly attributed to inappropriate warm-up, muscle fatigue, and muscle imbalance [8].

Soccer-related injuries are associated with both non-modifiable factors, such as sex and age, and modifiable factors, such as those that can be improved through programs that influence force, balance, and flexibility. Although both sets of factors interact and are risk determinants [13–15], professional players stop participating in soccer because of many modifiable causes [16]. The evaluation and implementation of preventive soccer training routines are essential, as injuries are associated with expensive treatment and prolonged withdrawal duration [5, 16].

The FIFA 11+ injury prevention program was developed in 2006 to address this matter, under the leadership of the FIFA Medical Assessment and Research Centre and in collaboration with the Oslo Sports Trauma Research Center and the Santa Monica Orthopaedic and Sports Medicine Center. The program comprises a complete warm-up procedure aimed at injury prevention in soccer players. It includes 15 structured exercises, is available as printed material or online, and is easily executed [15]. The exercises consist of core stabilization, eccentric thigh muscle training, proprioceptive training, dynamic stabilization, and plyometric exercises, all performed with proper postural alignment.

Program effectiveness was confirmed by various studies involving female and male players that revealed significant decreases in the incidence of non-contact injuries. The program was initially designed for amateur soccer; however, several studies demonstrated its effectiveness for other sports such as basketball [17].

The program is composed of 3 stages, with 15 exercises following a specific sequence. It is essential that the correct techniques are used, with emphasis on appropriate posture and body control, including leg alignment, knee positioning over the foot tip, and smooth landings (Table 1) [18]. The program is based on performing warm-ups at least twice a week [3]. Studies also indicated that a qualified trainer and medical monitoring are factors that influence the effectiveness of the FIFA 11+ program [19]. Furthermore, a period of at least 10–12 weeks is required to obtain results.

**Table 1:** Exercises and repetitions of the FIFA11+ program

Exercise	Repetitions
**I. Running exercises, 8 minutes (starting warming up, in pairs; Path consists of 6-10 pairs of parallel cones)**	
Running Straight Ahead	2
Running Hip Out	2
Running Hip In	2
Running Circling Partner	2
Running Shoulder Contact	2
Running Quick Forwards and Backwards	2
**II. Strength, plyometrics, balance, 10 minutes**	
**The Bench:**	
Level 1: static	3×20-30 sec
Level 2: alternate legs	3×20-30 sec
Level 3: one leg lift and hold	3×20-30 sec
**Sideways Bench:**	
Level 1: static	3×20-30 sec (each side)
Level 2: raise and lower hip	3×20-30 sec (each side)
Level 3: with leg lift	3×20-30 sec (each side)
**Hamstrings**	
Level 1: Beginner	3-5
Level 2: Intermediate	7-10
Level 3: Avanced	12-15
**Single-leg Stance**	
Level 1: hold the Ball	2×30 sec
Level 2: throwing ball with partner	2×30 sec
Level 3: test your partner	2×30 sec
**Squats:**	
Level 1: with toe raise	2×30 sec
Level 2: walking lunges	2×30 sec
Level 3: one leg squats	2×30 sec (each leg)
**Junping**	
Level 1: vertical jumps	2×30 sec
Level 2: lateral jumps	2×30 sec
Level 3:box jumps	2×30 sec
**III. Running Exercises, 2 minutes (End of heating)**	
Running across the pitch	2
Running bouding	2
Running plant and cut	2

This systematic review aimed at investigating the effectiveness of the FIFA 11+ program in preventing injuries in soccer players of both sexes aged >13 years by analyzing randomized clinical trial studies in the literature. This is the first systematic review to address the subject by exclusively using randomized clinical trials.

## Methods

**Fig. 1:**
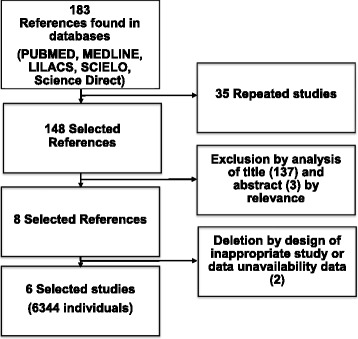
Organization chart of the selection of articles. PRISMA-2015 Protocol

This study was conducted using the PRISMA Statement 2015 (http://www.prisma-statement.org) [20]. The following databases were used: PubMed, MEDLINE, LILACS, SciELO, and ScienceDirect. The following keywords were searched: “FIFA,” “injury prevention,” and “football.” The research aimed at finding studies that reported on the effectiveness of the FIFA 11+ program for injury prevention in soccer players of both sexes aged >13 years. In the database search, 183 studies were found, of which 11 had a relevant title and only 8 were selected through their abstracts; 6 remained after text review through analysis of data availability and study design (Fig. 1).

The inclusion criteria were randomized clinical trial studies that analyzed relationships between the FIFA 11+ program and injury prevention in soccer players and studies published in Portuguese, English, Spanish, and French. Studies were evaluated using the Jadad scale [21]; all the studies scored >3. Only articles published between 2006 and 2016 were selected, based on the launch year of the program. Database searches were performed until March 14, 2016. The exclusion criteria were studies without randomized clinical trials, those that contained inadequate descriptions or incomplete data, those without soccer-playing populations, or those that associated the FIFA 11+ program with injury occurrence. The inclusion and exclusion criteria were sorted according to PICO (Patient, Intervention, Comparison, Objectives) (Table 2) [22].

**Table 2:** Inclusion criteria according to the acronym PICO

PICO	
Indicators	Results according to PICO
Project	Clinical Trials
Population	Participants (male and female), without restriction at a certain age (adolescents, professional and amateur players)
Intervention	FIFA11+
Comparisons	Conventional or without the FIFA 11+ warm-up program
Measures of Results	Injury/incidence rates

The chosen measure of association was relative risk (RR). Analyses were conducted using a random effects model, as studies presented significant variations among ages and training frequencies in addition to sex-related differences within the selected samples. Heterogeneity was evaluated using *τ* ^2^ and *I* ^2^ parameters, along with a critical analysis of the design and methodology. The risk of publication bias was analyzed using a funnel plot. All analyses were conducted using Review Manager 5.2v software (The Cochrane Collaboration, 2012).

## Results

The analysis included 6 studies performed in different countries, with 3 in Europe [23–25], 2 in North America [26, 27], and 1 in Africa [28]. The samples in 3 studies were composed of male players [23, 27, 28], and those in the other 3 were composed of female players [24–26]. All studies were randomized clinical trials that evaluated the effects of the FIFA 11+ program on injury prevention.

**Fig. 2:**
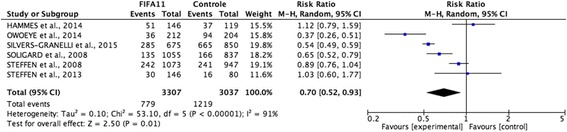
Analysis of the six independent samples, relating to the risk of injury in patients with different injury prevention programs

The total sample consisted of 6,344 players, of which 3,307 (52%) belonged to the intervention group (IG) and 3,037 (48%) belonged to the control group (CG). The IG had 779 injuries, while the CG had 1,219 injuries. Therefore, we can conclude that the FIFA 11+ program is effective for preventing injuries in soccer players, as its use led to a 30% reduction in injury occurrences, with an estimated RR of 0.70 (95% confidence interval [CI], 0.52–0.93; p = 0.01; Fig. 2).

The selected studies (through the described methodology) were submitted to a heterogeneity analysis using the Higgins and Thompson *I* ^2^ test [29, 30], which yielded an *I* ^2^ value of 91%. The inconsistency among the studies is probably due to sampling and methodology differences (Fig. 2).

Furthermore, heterogeneity was estimated using restricted maximum likelihood estimation (*τ* ^2^ = 0.10) and a chi-square test (χ^2^ = 53.10; p < 0.001), which also confirmed the heterogeneity among the studies and distortions in their distributions (Fig. 2).

Based on the results, we decided to investigate the heterogeneity due to the scarcity of references in the literature and the importance of the subject. A thorough analysis of references revealed that the heterogeneity was related to clinical factors inherent to the sample, clinical characteristics of the studies, and methodological heterogeneity.

A forest plot was used for result interpretation (Fig. 2), where the studies by Owoeye et al., 2014, Silvers-Granelli et al., 2015, Soligard et al., 2008, and Steffen et al., 2008, remained to the left of the vertical line, demonstrating that injuries were more likely to occur in the CG. Meanwhile, those by Hammes et al., 2014, Steffen et al., 2008, and Steffen et al., 2013 remained to the right, indicating that the results of the FIFA 11+ program were not significant. However, the outcome represented by the diamond graph remained to the left, indicating that the program is effective for injury prevention.

The consistent results of the analysis of lower limb injuries are highlighted by the RR of 0.70 (95% CI, 0.53–0.93; p = 0.02). In addition, the risk of moderate/severe injuries was analyzed in 5 studies that contained this information and revealed consistent RR results of 0.69 (95% CI, 0.54–0.88; p = 0.003).

**Fig. 3:**
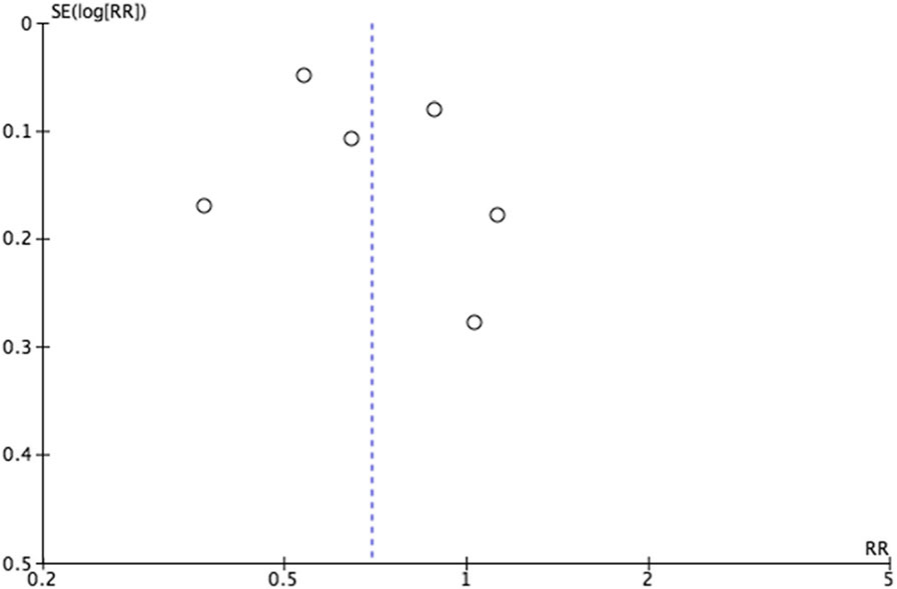
Evaluation of publication bias, showing homogeneity

Finally, this study did not show evidence of publication bias. Analysis was performed using a funnel plot (Fig. 3).

## Discussion

The FIFA 11+ injury prevention program has been recommended and adopted worldwide, owing to its effectiveness and easy application. The main finding of this study was that the program reduced injury risk by 30% (RR 0.70; 95% CI, 0.52–0.93). This result corroborates data from other studies regarding the effectiveness of the FIFA 11+ program for injury prevention in soccer players. One example is a cohort study performed in the USA in 2013 [31], which evaluated the protective effect of the program in male soccer players aged 18–25 years. The study adopted the first stage as the control and the second stage as the intervention evaluation and observed an RR reduction of approximately 72% (RR, 0.28; 95% CI, 0.09–0.85).

Soligard et al. (2010) obtained similar results when they evaluated the same parameters in female soccer players aged 13–17 years. The program was applied 1.3 times a week for 10 months and was associated with a 46% lower risk of injuries in the IG (odds ratio, 0.54; 95% CI, 0.33–0.87). In the same context, a systematic review published in 2014 [19] analyzed cohort and control studies and reported a 30–70% decrease in the occurrence of injuries in soccer players of both sexes aged >13 years. Another systematic review conducted by Mayo et al. (2014) included clinical and cohort studies and showed 33% and 57% reductions in injury occurrence, respectively. Thus, this meta-analysis also used studies that showed the effectiveness of the FIFA 11+ program (Table 3).

**Table 3:** Main aspects of references used on the revision

Authors/Year of publication	Methodological design	Number of subjects (N)	Groups Comparison	Intervention Protocol	Main results
Hammes et al., 2014 [22]	Randomized Clinical Trial	265	Intervention Group (n=146) Age: 45 ± 8 years old. Group Control (n=119) Age: 43 ± 6 years old. Gender: Male	Duration: 9 months Frequency: 1 time a week	No significant difference was found between the Intervention Group and the Control Group in the global incidence of lesions (IRR: 0.91 (0.64-1.48), p = 0.89) Only serious injuries reached significance Statistic with higher incidence in the Group Control (IRR: 0.46 (0.21-0.97), p = 0.04).
Owoeye et al., 2014 [27]	Randomized Clinical Trial	416	Intervention Group (n= 212) Group Control (n= 204) Age: 14-19 years old Gender: Male	Duration 6 months Frequency: 1 times a week	The FIFA11 + program significantly reduced the overall injury rate in the Intervention Group by 41% [RR = 0.59 (95% CI: 0.40-0.86; p = 0.006)] and lesions in lower limbs 48% [RR = 0.52 (95% CI: 0.34-0.82; p = 0.004)]. The FIFA11 + program is effective in reducing injury rates in young male soccer players.
Silvers-Granelli et al., 2015 [26]	Randomized Clinical Trial	1525	Intervention Group (n=675) Group Control (n=850) Age: 18-25 Gender: Male	Duration: 5 months Frequency: 1 time a week	In group Control, 665 injuries (mean ± SD, 19.56 ± 11.01) were reported by 34 teams, which corresponded to an incidence rate (IR) de 15.04 lesions per 1000 exposure time. In the Intervention Group, 285 lesions (mean ± SD, 10.56 ± 3.64) were reported by 27 teams, which corresponded to an IR of 8.09 lesions per 1000 exposure time. Total days lost due to injury were significantly higher for group control (Mean ± SD, 13.20 ± 26.6 days) than for the Intervention Group (mean ± SD, 10.08 ± 14.68 days) (P = 0.007). There was no difference for the loss of time due to an injury based on the field type (P = 0.341). FIFA11 + significantly reduced injury rates by 46.1% and decreased injury time loss, 28.6% in competitive male collegiate football player (rate ratio, [95% CI 49-, 59]), 54, P <0.0001.
Soligard et al., 2008 [24]	Randomized Clinical Trial	1892	Intervention Group (n=1055) Group Control (n=837) Age 15,4±0,7 Gender: Female	Duration: 8 months Frequency: 2 a 5 times a week	In the intervention group, there was a significantly lower risk of injury in general (RR = 0.68, 95%; CI 0.48-0.98), lower risk of overuse / fatigue injuries (RR = CI 0.47, 95% 0.26-0.85) and lower risk of serious injury (RR = 0.55, 95% CI 0.36-0.83) compared to the control group). The FIFA11 + program is effective in reducing injury rates in young female gender football players.
Steffen et al., 2013 [25, 27]	Randomized Clinical Trial	226	Intervention Group (n=146) Group Control (n=80) Age:13-18 years old Gender: Female	Duration: 4.5 months Frequency: 2-3 times a week	Compared to players with low adherence, players with high adherence to FIFA 11+ had a 57% lower risk injury (RR = 0.43; 95% CI 0.19-1.00). However, after adjustment for covariables, this difference between groups was not statistically significant (RR = 0.44; 95% CI 0.18-1.06)
Steffen et al., 2008 [23]	Randomized Clinical Trial	2020	Intervention Group (n=1073) Group Control (n=947) Age 13 – 17 years old Gender: Female	Duration: 8 months Frequency: 1 time a week	There was no difference in the overall lesion rate between the intervention (3.6 lesions / 1000 h, CI: 3.2-4.1) and group Control (3.7, Cl 3.2-4.1; RR 51.0, CI 0.8-1.2, P 50.94) or in the incidence of any type of injury.

Owoeye et al. [27] studied Nigerian players aged 14–19 years (n = 416, IG: 212, CG: 204) for 6 months and found that the FIFA 11+ program was effective, with a global injury reduction rate of 41% (RR, 0.59; 95% CI, 0.40–0.86; p = 0.006) during the evaluation period. Silvers-Granelli et al. [26] applied the program for American players of the National Collegiate Athletic Association and observed a 46.1% reduction in the injury rate (RR, 0.54; 95% CI, 0.49–0.59; p < 0.0001; number needed to treat, 2.64). Finally, Soligard et al. [24], who were the first to test the FIFA 11+ program, performed a randomized clinical trial with 1,892 female Norwegian players aged 13–17 years (IG: 1,055, CG: 837). The FIFA 11+ program was applied for 8 months, and a 32% reduction in injury incidence was observed (RR = 0.68; 95% CI, 0.48–0.98).

The literature presents a few studies that suggest the ineffectiveness of the use of the FIFA 11+ program in decreasing the injury rate, which highlights the need for an improved understanding of this subject. However, this dichotomy is possibly a result of the lack of a specific program protocol.

Analysis of the results of the systematic review showed increased heterogeneity (*I* ^2^ = 90%). We decided that a relevant approach would be to address this heterogeneity by identifying its main points in order to better understand factors that may interfere with the effectiveness of the program and to propose solutions.

A thorough reference analysis attributed the heterogeneity to clinical factors inherent to the sample, such as sex, age, body mass index (BMI), and clinical characteristics of the injuries. Furthermore, methodological heterogeneity may occur because of the lack of a protocol, type of warm-up adopted by the CG, non-blinded trainers, differences in capacity among training teams, and technical managers, as well as study frequency and duration.

In this review, the sample ages typically ranged from 13 to 25 years old, though the study conducted by Hammes et al. [22] included individuals aged >40 years (IG: 42.5 years old; CG: 43.1 years old). This factor is extremely relevant owing to the increased articular degeneration inherent to the aging process; furthermore, age also affects attitude and behavior during sports practice, physical resistance, and circumstances under which a soccer game is played [32, 33]. In this sense, player maturity is related to a higher commitment level and greater exercise awareness [24]. On the other hand, advanced age was identified as an injury risk factor in men aged >28 years and women aged >25 years [34]. It is worth emphasizing the effect of age on injury profiles, as younger athletes display more aggressive behavior while playing sports. This factor, which is associated with lower motor coordination in teenagers aged 14–16 years, explains the occurrence of higher contusion-type injuries in the lower limbs [35]. However, training is advantageous for young players, considering that they have not yet developed the bad habits of experienced players, which may ensure more correct exercise execution [25].

Another important factor was BMI, which is composed of non-modifiable (height) and modifiable factors (weight). Hammes et al. [23] reported a BMI suggestive of overweight for both groups (IG: 27; CG: 26,1). Some analyzed studies did not report BMI [24–27], while others presented normal values [28], which hindered analysis. This index modification (overweight or underweight) is related to higher injury occurrence, as overweight suggests less physical conditioning and, consequently, higher articular wear due to overload. On the other hand, underweight is related to reduced muscle mass and decreased ability to stabilize articulations during the game [16, 35].

Sex was another relevant clinical factor. Three studies analyzed men [23, 27, 28], and 3 analyzed women [24–26]. The literature presents clear evidence of higher overall injury rates in men [36]. However, women tend to have more ligament injuries [37, 38] and fewer muscle injuries than men [39, 40]. This injury profile may be explained by hormonal factors, especially those associated with sex, which are linked to anterior cruciate ligament injuries [41, 42].

Clinical aspects were also evaluated, and important differences were observed, which may have affected the heterogeneity of the results. Initially, emphasis was placed on the intrinsic subjectivity of injury categorization, even though all studies had declarations of consensus on the injury definitions and data collection procedures used in soccer studies [43] for evaluating player injuries. This means that the concept of injury and its categorization are subjective, both from the examiner’s and patient’s perspectives. This subjectivity may have been increased in the studies where injury was not evaluated by a qualified professional [23] and was diagnosed by the trainer or player.

Another relevant factor was the analyzed player type, as most were amateurs who are more susceptible to injuries because their technical abilities are inferior to those of professionals. In addition, professional players are more likely to adopt prevention programs [24]. Comparisons revealed that amateurs and professionals are more frequently injured during training and during the game, respectively. In addition, less severe injuries occur in professionals, whereas moderate and severe injuries are prevalent in amateurs [44].

Several studies used weekly monitoring [25, 27, 28], whereas other studies used monthly monitoring. Still other studies performed monitoring on demand [26]. Clearly, reliable results were more likely obtained by those who performed more frequent monitoring. When trainers questioned the reports, the programs and monitoring were more effectively conducted. This methodology may also have contributed to reductions in partial or incorrect reporting, which was present in most of the studies.

Attention to proper trainer monitoring and data recording was found to be an essential factor, as the trainers were present in some studies [23, 26, 27], while in other studies, this spare-time role was filled by parents [25] or “advisory players” [23] who complained about the overload inherent to this activity. Such overload combined with trainers lacking knowledge regarding randomization explained the abandonment of the CG in several studies, as many trainers felt discouraged because they were not selected for the IG or were simply not available for data reports.

Methodology was also associated with relevant factors. As previously mentioned, the FIFA 11+ program is easily applicable; however, effectiveness is only obtained if exercises are performed within the existing standards. On the other hand, homogeneity in program application was absent among different reference groups, which affected the results obtained through the combined analysis. A standard protocol for the warm-up program application was nonexistent; thus, application in some studies was according to FIFA recommendations, i.e., 2 or 3 times a week [25, 26], while others used 1-week intervals [23, 24, 27, 28]. Furthermore, differences were observed in the study period duration, which varied between 4.5 and 9 months. All these factors may have affected the results, which may be more reliable in the groups that applied the program using the recommended frequency; weekly evaluations may not have revealed the true program effectiveness. Therefore, it is important to consider that the effectiveness of warm-up programs depends on long-term factors such as the development of muscular strength for protecting, supporting, and stabilizing skeletal articulations [16]. Thus, studies with shorter durations may not have allowed sufficient time for the development of appropriate muscular strength. The minimum duration that is necessary according to the program recommendation is 10–12 weeks, if applied using the appropriate frequency [18].

Methodological differences were also identified in terms of the capacity and randomization knowledge of the trainers. All results revealed that program assessors were not blinded; correct FIFA 11+ program application would be impossible without proper knowledge. All the studies used support materials such as DVDs, posters, and online information; only two studies used a training workshop [27] or a 3-hour course conducted by the Oslo Sports Trauma Research Center [25]. After these sessions, however, trainers could not count on improvement sessions or monitoring to guarantee conformity in the application of the FIFA 11+ program. It is worth noting that trainers are the key to promoting injury prevention, as they are responsible for regular and correct exercise execution.

Few studies verified exercise similarities among groups [25, 27], which may have affected the results. A CG performing similar exercises may have compromised data reliability. Furthermore, even with proper randomization and group blinding, information about the FIFA 11+ program is easily found online, which could have compromised the study blinding.

Thus, it is important to emphasize that soccer is one of the most popular sports worldwide; however, it carries a significant risk of injuries, especially in the lower limbs. These injuries are mainly related to modifiable factors, which corroborates the critical role played by warm-up programs. Accordingly, these programs should be easily applicable and involve all soccer players, which is consistent with the proposal of the FIFA 11+ program. Thus, new research on this subject is warranted and must follow the recommendations of the FIFA 11+ program to determine its effectiveness based on a specified utilization period (frequency and duration) and quality of exercise performance.

The effectiveness of the program is evident in committed players who are supported by training and health teams. The ineffectiveness presented by some studies is related to the lack of commitment to a program venue and an inappropriate application period or frequency due to the lack of motivation by trainers or players involved in the intervention.

The fact that the FIFA 11+ program was launched in 2006 is a limitation, as it is recent and there are few literature references. Moreover, the few studies available in the literature did not follow a specific protocol or provide a venue for the warm-up programs. These factors were also observed in references analyzed by this study, where important clinical differences were identified within the sample and were important injury-determining factors. In addition, the characteristics of the clinical studies may have generated categorization errors or modifications in the injury incidence among players. The methodological approach adopted by studies also presented limitations, which may have affected results and contributed to possible program ineffectiveness resulting from application errors.

## Conclusion

The FIFA 11+ warm-up program is effective for preventing injuries in soccer players of both sexes aged >13 years.
